# A new technique to salvage myocardium following the failure of thrombus aspiration in acute myocardial infarction: a case report

**DOI:** 10.1186/s12872-018-0951-9

**Published:** 2018-12-10

**Authors:** Daoyuan Si, Guohui Liu, Yaliang Tong, Cheng Zhang, Yuquan He

**Affiliations:** Department of Cardiology, China-Japan Union Hospital of Jilin University, Jilin Provincial Engineering Laboratory for Endothelial Function and Genetic Diagnosis of Cardiovascular Disease, Jilin Provincial Cardiovascular Research Institute, Xiantai Street NO.126, Changchun, Jilin, China

**Keywords:** Acute myocardial infarction, Thromboembolism, Aspiration thrombectomy, Left main coronary artery

## Abstract

**Background:**

The failure of aspiration thrombectomy may negatively impact outcomes in patients with acute myocardial infarction (AMI), but the available options are limited.

**Case presentation:**

A 41-year-old man with chest pain for 2 h presented with ST-segment elevation myocardial infarction. Coronary angiography revealed a large filling defect extending from the distal left main (LM) coronary artery into the proximal left circumflex (LCX) coronary artery. The whole thrombus moved and occluded the proximal left anterior descending (LAD) artery, while the guidewire crossed the lesion. Dedicated manual aspiration thrombectomy (MAT) and balloon dilation failed to reduce thrombus burden. We considered thrombus extraction as impossible when it moved forward to occlude the middle LAD. To reduce infarct size, a new balloon-pushing technique was successfully performed to move the thrombus to the terminal LAD based on the actual condition of the LAD. The final angiogram demonstrated no stenosis in the LM artery and stent deployment was not performed. A 1-week follow-up coronary angiography revealed the complete resolution of thrombus and flow restoration in the left coronary artery. Intravascular ultrasound (IVUS) showed nonsignificant residual stenosis of the LM artery. No adverse events occurred during a 12-month follow-up period.

**Conclusion:**

This case suggests that the new balloon-pushing technique is a useful remedy if repeated MAT fails during AMI.

## Background

Although thrombus aspiration in acute myocardial infarction (AMI) did not lead to clinical benefits according to two recent two large randomized clinical trials [1, 2], thrombus aspiration during the primary percutaneous coronary intervention (PCI) might decrease the risks of stent thrombosis and reinfarction [1, 3]. Thrombus aspiration remains in use in numerous patients with AMI because of its appealing effect regarding the reduction of thrombus burden and no-reflow phenomena [4]. However, the available options are limited when manual aspiration thrombectomy (MAT) fails during primary PCI, especially among patients with thromboembolism. Here, we report a case of a successful myocardium salvage through a new balloon-pushing technique following the failure of repeated MAT in a patient receiving primary PCI.

## Case presentation

**Fig. 1:**
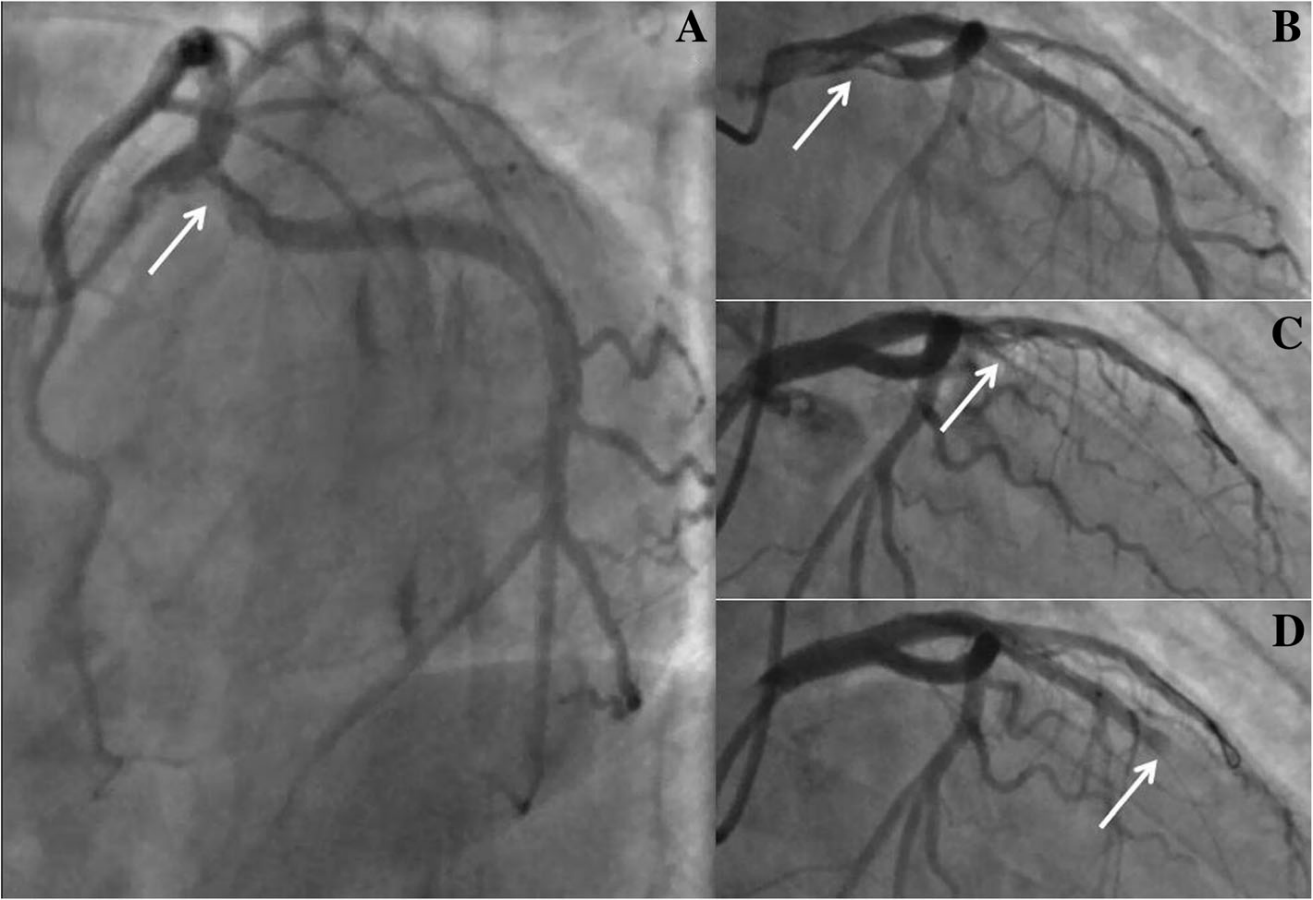
Angiography and movement of thrombus. **a** and **b** Initial left coronary angiogram demonstrating a filling defect (white arrow) in the left main coronary artery extending into the left circumflex coronary artery. **c** The whole thrombus (white arrow) moved into the proximal left anterior descending artery while the guidewire crossed the lesion. **d** The thrombus (white arrow) moved toward the middle site

**Fig. 2:**
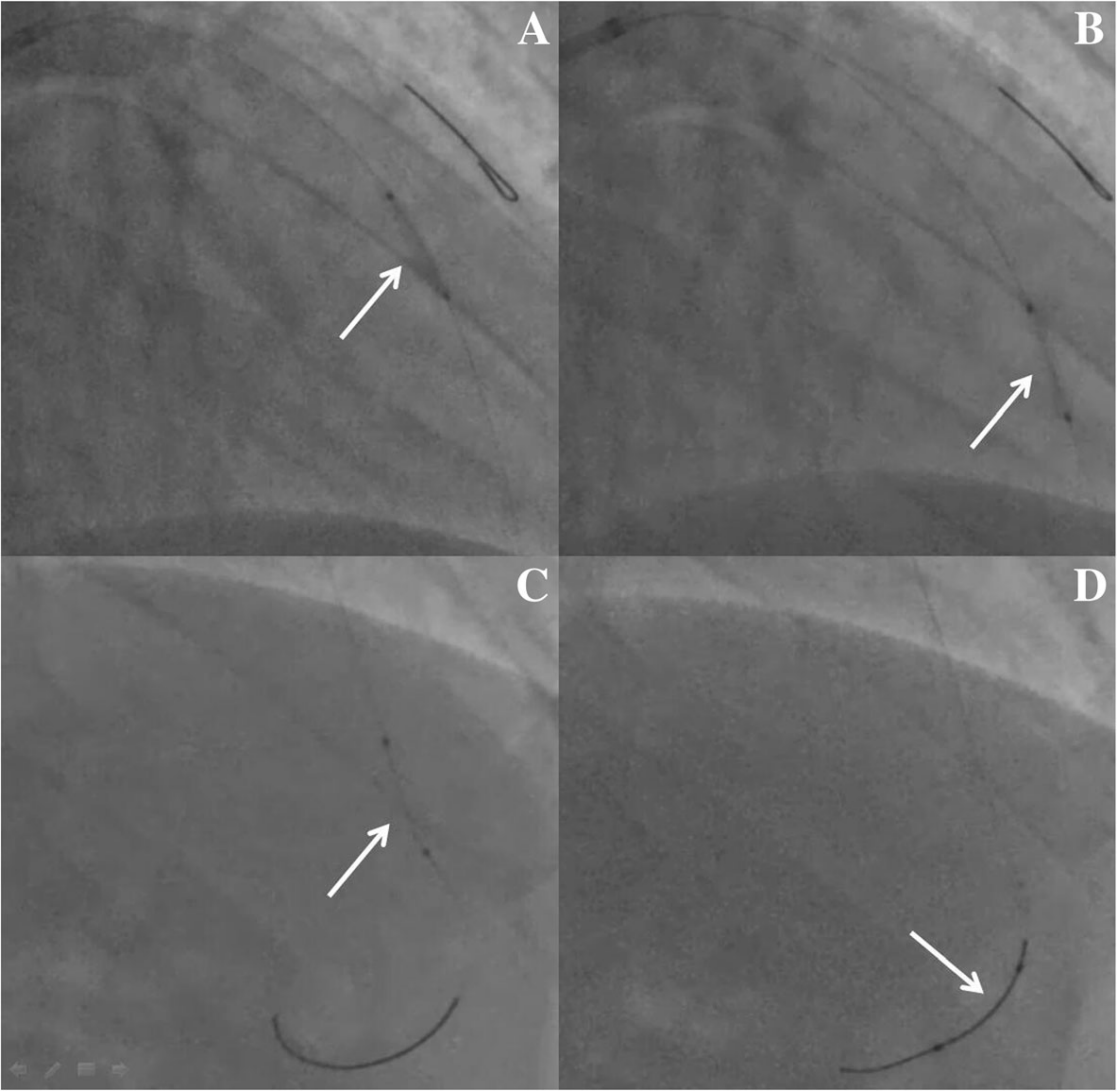
Balloon pushing technique. **a** and **b** The dilated balloon (white arrow) pushing the thrombus toward the distal left anterior descending artery (LAD). **c** and **d** The undilated balloon (white arrow) pushing the thrombus to the terminal LAD

**Fig. 3:**
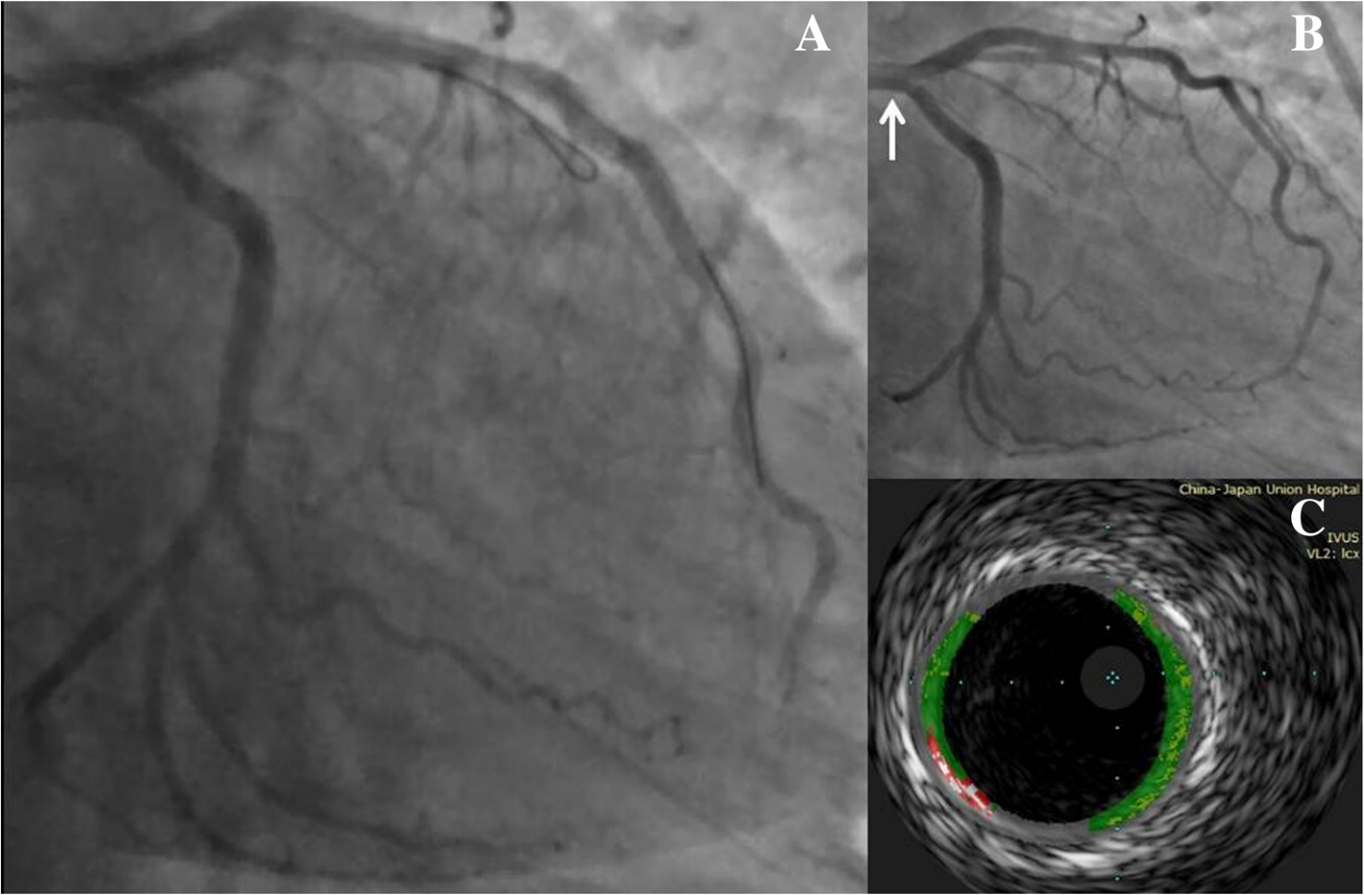
Final result and follow-up. **a** The final angiogram showed that only the terminal left anterior descending artery (LAD) was occluded by the thrombus. **b**. No angiographic signs of the residual thrombus in LAD were revealed in follow-up angiography. **c** Intravascular ultrasound (IVUS) showed non-significant residual stenosis of the left main coronary artery

A 41-year-old male heavy smoker with no specific medical history was admitted to our hospital with severe chest pain lasting 2 h. His blood pressure was 130/80 mmHg on admission, and he presented with no laterality in the upper extremities. Electrocardiography on arrival showed ST-segment elevation in leads II, III, and aVF. The cardiac troponin I was 0.68 ng/ml. He was diagnosed with inferior ST-segment elevation myocardial infarction (STEMI) in Killip Class I and immediately brought to the cardiac catheterization laboratory. An emergency coronary angiography revealed a large filling defect extending from the distal LM artery into the proximal LCX artery (Fig. 1a and b). Otherwise, no significant lesions were found and thrombolysis in myocardial infarction (TIMI) III flows were observed in all coronary arteries. Therefore, PCI was performed using a 6Fr guiding catheter (EBU3.5, Medtronic). Unfortunately, while a 0.014-in. guidewire (Runthrough, Terumo) crossed the LM artery, the whole thrombus was extracted from the proximal LCX artery and pushed into the LAD artery. The proximal LAD artery was completely occluded by the thrombus (Fig. 1c). After crossing the lesion in the LAD artery with the guidewire (Runthrough, Terumo), thrombectomy was attempted several times using an aspiration catheter (Export, Medtronic), and the intracoronary administration of Glycoprotein IIbIIIa inhibitor was slowly infused. However, these treatments did not reduce the thrombus burden in the proximal LAD artery, and no visible thrombus was detected in the aspirate. Then, a 14-atm dilation of a semicompliant balloon (Ryujin 2.5 × 15 mm, Terumo) was performed in the lesion. However, the thrombus moved to the middle LAD artery with TIMI flow 0 (Fig. 1d). Following the failure of another attempt of aspiration using an Export catheter (Medtronic), thrombus extraction from the LAD artery was considered as impossible. The initial angiogram showed no stenosis in the LAD artery, which had diameters above 2.5 mm and above 1.5 mm at the distal and terminal sites, respectively. To reduce the infarct size, we decided to try a new technique by pushing the thrombus to the terminal LAD artery. A dilated balloon with 6 atm (Ryujin 2.5 × 15 mm, Terumo) was used to push the thrombus carefully toward the distal LAD artery (Fig. 2a and b). Furthermore, the contracted balloon (Ryujin 2.5 × 15 mm, Terumo) successfully pushed the thrombus to the terminal LAD artery (Fig. 2c and d). The final angiogram demonstrated no other significant stenosis except the embolization in the terminal site of the LAD artery (Fig. 3a); thus, stent deployment was not performed.

The patient was transferred to the coronary intensive care unit in a hemodynamically stable condition, while his ST elevation subsided. He was started on low-molecular-weight heparin in addition to regular aspirin, ticagrelor and rosuvastatin. Glycoprotein IIbIIIa inhibitor administration was continued for 24 h. The immunological screen results were unremarkable. A follow-up coronary angiography was performed 1 week later, which revealed the restoration of TIMI 3 flow and the complete resolution of thrombus in the left coronary artery (Fig. 3b). Intravascular ultrasound (IVUS) showed nonsignificant residual stenosis of the LM artery (Fig. 3c). Echocardiography revealed the left ventricular ejection fraction (EF) was 61% with a slight apex hypokinesis, and the bubble study showed a negative result. The patient had an uncomplicated recovery and was discharged after 2 days. No adverse events occurred during a 12-month follow-up period.

## Discussion and conclusions

LM artery thrombosis is rare and challenging in the context of AMI, with an estimated incidence of 0.8–1.7% [5]. The usual trigger is the fibrous cap rupture of an atherosclerotic plaque or plaque erosion without rupture. Other causes include coronary embolism, which has an incidence of 3% during AMI [6]. The pathophysiologic and anatomic substrates of coronary embolism are hypercoagulability, endothelial injury, blood stasis and anatomic predispositions such as patent foramen ovale and mitral stenosis [6]. However, no evidence of the above was found in the present case. Thus, the filling defect detected in the present case was estimated to be a thrombus formation, not an embolism; however, a 1-week follow-up IVUS did not reveal the absence of atherosclerotic plaque. Surface erosion might explain this finding because the angiogram demonstrated no residual stenosis in the LM artery, and the follow-up IVUS did not detect any sign of plaque rupture. As a consequence, additional balloon angioplasty or stent placement was not performed. Consistent with our case, several case series demonstrated that additional angioplasty and stenting might not be necessary if thrombectomy treatment results in the complete restoration of coronary flow without significant residual stenosis or signs of plaque disruption [7, 8]. A recent 1-year follow-up report within the EROSION study demonstrated that a majority (92.5%) of patients with acute coronary syndrome caused by plaque erosion managed with aspirin and ticagrelor without stenting remained free of major adverse cardiovascular events for ≤1 year [9]. Our case suggests that this concept is also appropriate for LM artery thrombosis. Following conservative enhanced antiplatelets treatment, repeat coronary angiography should be performed to check the thrombus resolution. Intravascular imageology might facilitate the evaluation of the residual plaque and the need for further intervention.

Several studies have not shown a substantial benefit regarding the routine use of MAT during primary PCI [1, 2, 10]. Of interest, aspiration thrombectomy was associated with a significant reduction in cardiovascular death in a subgroup of patients with large thrombus burden, suggesting that MAT is a valid option for certain patients [11]. MAT, which is also suggested by clinical guidelines, remains one of the most frequently used thrombectomy methods when intracoronary thrombi are encountered in the context of AMI. However, feasible alternative methods are limited when MAT failed during AMI, despite improvements in the MAT technique [12]. In our case, the thrombus was pushed to occlude the proximal LAD artery accidentally. Several MAT attempts failed to reduce the thrombus burden, and the condition deteriorated when the thrombus moved to the middle LAD artery. Thus, we considered it as impossible to remove the thrombus quickly during this primary PCI. The primary goal of primary PCI is the reperfusion of the infarcted myocardium. Because rapid and complete reperfusion could not be achieved in this unexpected scenario, we tried a new technique to minimize the infarct size. According to the anatomic characteristic of the LAD artery, we successfully pushed the thrombus to the terminal LAD artery using a balloon. A 1-week follow-up coronary angiography revealed the restoration of TIMI 3 flow and the complete resolution of the thrombus in the LAD artery following conservative antiplatelets treatment. Echocardiography also showed slight apex hypokinesis with normal EF.

The technical characteristics of this method are described below. First, we must ensure the distal portion of the target artery is normal; otherwise, the pushing process will cause additional injury to the pre-existing lesions, such as dissection of the coronary artery. Second, the diameter of the balloon selected should be smaller than that of the distal artery. Third, if the angiography result is not satisfying after the thrombus has been pushed to the distal site via the dilated balloon, then a contracted balloon can be used to push the thrombus to the terminal site. The key point of this technique is switching the proximal thrombosis to the terminal thrombosis, minimizing the infarct size when it cannot be extracted. To the best of our knowledge, our report is the first to describe this alternative method of MAT in primary PCI.

This new balloon pushing technique proved to be effective and feasible in the current case. This procedure might represent a simple and viable remedy for patients with AMI unresponsive to conventional treatment. Although this technique is limited by its inherent characteristics, our experiences provided a practical alternative method for thrombus reduction in cases of primary PCI.

## Abbreviations

AMI: Acute myocardial infarction
EF: Ejection fraction
IVUS: Intravascular ultrasound
LAD: Left anterior descending coronary artery
LCX: Left circumflex coronary artery
LM: Left main coronary artery
MAT: Manual aspiration thrombectomy
PCI: Percutaneous coronary intervention
STEMI: ST-elevation myocardial infarction
TIMI: Thrombolysis in myocardial infarction
